# Meteorological factors and childhood diarrhea in Peru, 2005–2015: a time series analysis of historic associations, with implications for climate change

**DOI:** 10.1186/s12940-021-00703-4

**Published:** 2021-02-26

**Authors:** Miranda J. Delahoy, César Cárcamo, Adrian Huerta, Waldo Lavado, Yury Escajadillo, Luís Ordoñez, Vanessa Vasquez, Benjamin Lopman, Thomas Clasen, Gustavo F. Gonzales, Kyle Steenland, Karen Levy

**Affiliations:** 1 Gangarosa Department of Environmental Health, Rollins School of Public Health, Emory University, 1518 Clifton Road, Atlanta, GA USA; 2 Department of Public Health, Administration, and Social Sciences, School of Public Health and Administration, Universidad Peruana Cayetano Heredia, Avenida Armendáriz 445, Miraflores, Lima, Peru; 3 Servicio Nacional de Meteorología e Hidrología del Perú (SENAMHI; National Meteorology and Hydrology Service of Peru), Jirón Cahuide 785, Jesús María, Lima, Peru; 4 National Center for Epidemiology, Prevention and Control of Diseases, Ministerio de Salud (MINSA; Ministry of Health), Avenida Salaverry 801, Jesús María, Lima, Peru; 5 Department of Biological and Physiological Sciences, Faculty of Sciences and Philosophy, Universidad Peruana Cayetano Heredia, Avenida Honorio Delgado 430, San Martín de Porres, Lima, Peru; 6 Department of Epidemiology, Rollins School of Public Health, Emory University, 1518 Clifton Road, Atlanta, GA USA; 7 Department of Environmental and Occupational Health Sciences, School of Public Health, University of Washington, 1959 NE Pacific Street, Seattle, Washington USA

**Keywords:** Diarrhea, Climate change, Temperature, El Niño, Drinking water

## Abstract

**Background:**

Global temperatures are projected to rise by ≥2 °C by the end of the century, with expected impacts on infectious disease incidence. Establishing the historic relationship between temperature and childhood diarrhea is important to inform future vulnerability under projected climate change scenarios.

**Methods:**

We compiled a national dataset from Peruvian government data sources, including weekly diarrhea surveillance records, annual administered doses of rotavirus vaccination, annual piped water access estimates, and daily temperature estimates. We used generalized estimating equations to quantify the association between ambient temperature and childhood (< 5 years) weekly reported clinic visits for diarrhea from 2005 to 2015 in 194 of 195 Peruvian provinces. We estimated the combined effect of the mean daily high temperature lagged 1, 2, and 3 weeks, in the eras before (2005–2009) and after (2010–2015) widespread rotavirus vaccination in Peru and examined the influence of varying levels of piped water access.

**Results:**

Nationally, an increase of 1 °C in the temperature across the three prior weeks was associated with a 3.8% higher rate of childhood clinic visits for diarrhea [incidence rate ratio (IRR): 1.04, 95% confidence interval (CI): 1.03–1.04]. Controlling for temperature, there was a significantly higher incidence rate of childhood diarrhea clinic visits during moderate/strong El Niño events (IRR: 1.03, 95% CI: 1.01–1.04) and during the dry season (IRR: 1.01, 95% CI: 1.00–1.03). Nationally, there was no evidence that the association between temperature and the childhood diarrhea rate changed between the pre- and post-rotavirus vaccine eras, or that higher levels of access to piped water mitigated the effects of temperature on the childhood diarrhea rate.

**Conclusions:**

Higher temperatures and intensifying El Niño events that may result from climate change could increase clinic visits for childhood diarrhea in Peru. Findings underscore the importance of considering climate in assessments of childhood diarrhea in Peru and globally, and can inform regional vulnerability assessments and mitigation planning efforts.

**Supplementary Information:**

The online version contains supplementary material available at 10.1186/s12940-021-00703-4.

## Background

Global temperatures are projected to rise by 2 °C or more by the end of the twenty-first century [1, 2], which is expected to impact the incidence of diseases caused by pathogens that can survive or multiply in the environment, including diarrhea [3, 4]. A large percentage of the burden of climate change-related morbidity is expected to be borne by children [5], who have unique vulnerabilities to climate change [6].

The World Health Organization (WHO) projects an annual increase of approximately 50,000 child diarrheal deaths worldwide in 2050 attributable to climate change [7]. In Peru, the rate of childhood clinic visits for diarrhea has been declining since 2005 [8]. However, Peru and other countries that have made substantial gains toward lowering diarrhea morbidity may have these trends slowed or reversed as climate change and increasing temperatures bring additional challenges to reducing diarrheal disease morbidity [2]. The El Niño phenomenon may also contribute to such challenges: research in Peru has shown significant increases in diarrhea cases during El Niño events [8–15]. Climate change is expected to increase the frequency and intensity of El Niño periods [16]; in recent decades, El Niño events have been intensifying in the Eastern Pacific, near the coast of Peru [17].

Estimations of increased diarrhea mortality as a result of climate change are based on systematic reviews that have demonstrated a positive association between temperature and diarrhea cases, although not all diarrheagenic pathogens display this relationship [7, 18, 19]. Diarrhea caused by rotavirus, historically a leading cause of childhood diarrhea, is more common at lower temperatures [18, 20, 21]. In 2009, WHO announced a recommendation for rotavirus vaccine inclusion in national immunization programs [22]. Global rotavirus vaccination has since increased, with early studies showing large reductions in severe rotavirus cases [23]. As rotavirus cases are reduced through widespread vaccination, the overall positive association between temperature and diarrhea could strengthen, as cases of rotavirus diarrhea may be averted during cooler periods. Other studies have demonstrated rotavirus vaccination shifting or diminishing the seasonality of rotavirus infections [24–26] and that the association between meteorologic factors and rotavirus infections varies between settings with and without ongoing rotavirus vaccination [27].

While there have been many published works examining the relationship between temperature and diarrhea and how the seasonality of certain diarrheal illnesses may be modified by rotavirus vaccination [24–26], little attention has been given to how the relationship between temperature and childhood diarrhea may be modified or affected by other environmental variables such as access to improved drinking water. Establishing these historic relationships is important to climate change vulnerability and mitigation assessments [18, 28].

The objectives of this analysis were (1) to estimate the association between temperature and clinic visits for childhood diarrhea in Peru, accounting for El Niño, wet/dry seasons, and the introduction of rotavirus vaccination, and (2) to determine whether the temperature-diarrhea association varied by level of piped water access, hypothesizing that improved drinking water quality and availability might interrupt some of the pathways through which higher temperatures may increase diarrhea cases [19].

## Methods

The analysis utilized an ecologic study design in which the unit of observation was a province, and used an extensive secondary dataset compiled from governmental surveillance data, censuses and surveys, and meteorologic estimates.

### Geographic scope

Peru is comprised of 25 departments, which encompass 195 provinces, which are further sub-divided into districts. Each district belongs to a single province, allowing for aggregation of district data to the provincial level. Analyses were conducted for the 195 provinces of Peru: data from districts assigned to a new (196^th^) province in 2014 were analyzed with their former province. Province populations range from < 4000 residents (Purus province, Ucayali department) to > 7 million residents (Lima province); areas range from ~ 150 km^2^ (province of Callao) to > 67,000 km^2^ (province of Loreto) [29]. The choice of conducting the analysis at a province level, rather than at a district level, was based on small child populations in many districts; this meant a high proportion of weeks had no diarrhea cases in many districts. There were also difficulties in obtaining estimates of the child population and piped water access at a district level.

### Data sources and definitions

#### Diarrhea cases and rotavirus vaccine data

The Peruvian Ministry of Health (MINSA) collects obligatory weekly surveillance data on diarrhea visits from all public inpatient and outpatient clinics in Peru. Private clinics also send regular weekly reports if they opt into the reporting system. Diarrhea cases refer to patients presenting to a clinic with an increase in frequency of bowel movements (three or more bowel movements in 24 h), or in fluidity or volume of stool compared to usual, with onset within the past 2 weeks. Cases are aggregated by age group (< 1 year old, 1–4 years old, ≥5 years old), and assigned to the patient’s district of residence. We use the term “childhood diarrhea rate” to describe the rate of clinic visits for diarrhea in children under 5 years old. We analyzed cases reporting to clinics between January 5, 2005 and December 16, 2015. Weekly data on diarrhea were summed for each province.

MINSA also collects rotavirus immunization data. Rotarix®, which is administered to infants in two doses between approximately 6 weeks and 6 months of age, was added to the national immunization schedule in 2009. By 2010, most Peruvian infants were receiving both doses of the rotavirus vaccine [8]. Data were analyzed for the “pre-(rotavirus) vaccine era” (2005–2009) and “post-vaccine era” or “rotavirus vaccine era” (2010–2015).

#### Piped water and population

Data from the Peruvian national census (2007 and 2017) and SISFOH (El Sistema de Focalización de Hogares, the Household Focalization System conducted 2012–2013) were used to estimate the percentage of households in each province with access to piped water (households for which the primary drinking water source was water piped inside or outside of the home, but within the building area). The national censuses in 2007 and 2017 were used to estimate the annual population of children under 5 years old by province. Detailed methods of obtaining annual piped water and population estimates were described previously [8].

In order to classify provinces by piped water access across the 11-year study period, provinces were divided into three groups: (1) provinces that had consistently lower access than the rest of the country throughout the study period, (2) provinces that had consistently higher access, and (3) provinces that fit into neither category, namely that transitioned from lower to higher access throughout the study period. Low piped drinking water access provinces were defined as those in which < 60% of households had access to a piped water connection in all study years (2005–2015), or all but 1 year. High piped drinking water access provinces were those in which ≥60% of households had access to a piped water connection in all study years, or all but 1 year. Transitional provinces were those in which < 60% had access to piped water for at least two study years, but transitioned to higher access (≥60% for at least 2 years). The choice of cut-off for the categories was based on the median access level across all years and all provinces, to maximize the number of provinces in the high and low categories. In our previous research utilizing these data, we observed that provinces with higher levels of access to piped drinking water also tended to have higher levels of access to a toilet connected to the sewerage system [8]; therefore, in our primary analysis we considered only piped water access as a proxy for improved drinking water and sanitation.

#### Temperature data source

The Peruvian National Meteorology and Hydrology Service (SENAMHI) provided temperature data from the Peruvian Interpolation of SENAMHI’s Climatological and hydrological Observations temperature product (PISCOt). Temperature estimates were constructed utilizing data from 178 quality-controlled, gap-filled, and homogenized air temperature stations, remote-sensed data, and a set of topographic predictors [30]. PISCOt includes daily minimum temperatures (Tmin) and maximum temperatures (Tmax) at 0.1° gridded spatial resolution (each grid approximately 10 km × 10 km) for the country of Peru. The Tmin and Tmax values were averaged to create gridded daily mean temperature (Tmean) values.

### Construction of meteorological variables

#### Control for dry/rainy season

Provinces were classified into one of Peru’s three major geographic regions: the coast (departments in western Peru bordering the Pacific Ocean), the Peruvian Amazon (departments in eastern Peru), and the mountains (running north to south between the coast and Peruvian Amazon, along the Andes Mountains) [31]. June–August was considered the dry season in the mountain provinces and the Peruvian Amazon provinces. Coastal provinces receive very little rainfall, nevertheless have a drier season from June to November, which was classified as the dry season in analyses [32].

We did not consider precipitation measurements in this analysis. Rainfall patterns tend to be highly localized and more spatially variable than temperature estimates; use of typical rainfall datasets can lead to large bias in analyses of waterborne disease [33]. The association between rainfall and diarrhea is complex: though heavy rainfall can be associated with increased diarrhea, this relationship may depend on antecedent conditions [34, 35].

#### El Niño

The El Niño Southern Oscillation (“El Niño”), is a global pattern of climate variability associated with unusual warming of the Pacific Ocean near the equator, resulting in increased temperature and changes in precipitation in Peru [36]. The U.S. National Oceanic and Atmospheric Administration (NOAA) reports data on the Oceanic Niño Index (ONI), calculated using a standard 3-month mean of sea surface temperature anomalies in the Niño 3.4 region of the Pacific Ocean [37]. For each month in our study, we assigned the corresponding ONI value of the three-month running average in which the study month was the midpoint. For example, the ONI for February 2005 was assigned the ONI running average for January–March 2005. El Niño periods were defined using the ONI, with values in the ranges 0.5–0.9, 1.0–1.4, and ≥ 1.5 corresponding to weak, moderate, and strong El Niño events, respectively [38]. We compared months with a moderate or strong El Niño to months with a weak El Niño or no El Niño. Moderate/strong El Niño periods in the study were from October 2009 to February 2010 and May–December 2015.

#### Province-level temperature estimates

The gridded Tmax temperature values were averaged within each district boundary, giving a daily high temperature for each district. Province-level daily temperature estimates were estimated using population-weighted averages of the district-level temperatures, giving more weight to temperatures in more populous districts of the province. The same process was used to estimate province-level daily Tmean values.

After comparing three temperature variables for model fit (the weekly maximum of the daily high temperatures, the weekly mean of the daily high temperatures, and the weekly mean of the daily mean temperatures; Additional File 1), the weekly mean of daily high temperatures fit the data best and was selected as the main temperature exposure variable for the multivariable models described in this manuscript. An example of a time series displaying the weekly mean of daily high temperatures, including indications of dry seasons and El Niño events, is provided in Additional File 2.

### Statistical analysis

Data were compiled and cleaned in R 3.5.1 (R Foundation for Statistical Computing, Vienna, Austria) and analyzed using SAS 9.4 (SAS Institute, Inc., Cary, NC). We analyzed province-level weekly counts of clinic visits for diarrhea in children < 5 years using negative binomial generalized estimating equations (GEEs) with autoregressive correlation structures. We first examined whether temperature was associated with weekly childhood clinic visits for diarrhea in a model that included the following variables: the weekly mean of daily high temperatures, with distributed lags of 1, 2 and 3 weeks; an indicator for the rotavirus vaccine era; an indicator for dry/wet season (which varied by month and region of Peru); an indicator for moderate/strong El Niño events; a continuous variable for the study year (to account for secular trend); and a variable to control for province, to focus on week-to-week temperature changes within each province, while controlling for other unmeasured province-level factors. We also included an offset for provincial population (child population < 5 years).

To examine whether the association between temperature and childhood clinic visits for diarrhea differed between the pre- and post-vaccine eras, we ran the model above with additional terms for the interaction between each lagged temperature value and the rotavirus vaccine era term. All analyses were performed on all provinces combined, and also stratified by the three groups of access to piped water, in three separate models. As the definition of piped water access was fixed for each province for the study period, only a stratified analysis was performed to assess effect modification; piped water access was not considered as a main effect.

The exponentiated coefficients for the included terms were reported as estimates of incidence rate ratios (IRRs). In addition to the separate IRRs for each of the three lagged terms for temperature, the combined effect of temperature across all included lags (the exponentiated sum of the three coefficients) was also reported as an IRR estimate for a single exposure metric, referred to here as the “temperature-diarrhea association”.

#### Sensitivity analysis

Many provinces of Peru have low temperature variability, i.e.*,* temperature is relatively constant throughout the year. Hypothesizing that these provinces might contribute little to the temperature-diarrhea association, we conducted a sensitivity analysis limited to provinces with higher variability in daily maximum temperature.

To consider the variability in temperature throughout the year in each province, we constructed monthly average temperatures, which were the mean of the daily high temperatures in each month of the year, excluding data from El Niño periods. As an example, the January mean daily high for a province was an average of all daily high temperatures from any January in the study period (2005–2015), excluding any January during El Niño periods. Of the 194 provinces included in the analysis, 133 (68.6%) had less than a 3 °C difference between the mean temperature in the warmest month and coolest month (Additional File 3). These provinces were classified as having low temperature variability and were excluded in the sensitivity analysis models.

## Results

The analysis included data from 194 Peruvian provinces. Ocros province was excluded due to inconsistent diarrhea surveillance reporting. Data were analyzed from 1838 districts, with < 1% (18 districts) having any missing diarrhea reports. Descriptive province-level statistics are displayed in Table 1.

**Table 1:** Descriptive statistics, provinces of Peru, 2005–2015

	Median (IQR) among 194 provinces
**Annual clinic visits for diarrhea per 100 children < 5 years old**	
2005	28.8 (21.3–40.0)
2015	21.7 (15.4–31.4)
**Child population (< 5 years old, 2017 census)**	5219 (2454-10,358)
**Average daily high temperature**^**a**^**, °C**	
January	21.2 (18.4–27.8)
July	21.2 (18.9–24.7)
**Percentage of households with access to piped water**^**b**^	
2005	42.8% (21.0–61.7%)
2015	69.7% (58.5–78.5%)
	***N (%)***
**Piped water access group**^**c**^ **and geographic region**	**(*N*** **= 194 provinces)**
**Coastal provinces**	**49 (25.3%)**
Low piped water access	10 (20.4%)
Transitional piped water access	14 (28.6%)
High piped water access	25 (51.0%)
**Mountain provinces**	**114 (58.8%)**
Low piped water access	38 (33.3%)
Transitional piped water access	48 (42.1%)
High piped water access	28 (24.6%)
**Peruvian Amazon provinces**	**31 (16.0%)**
Low piped water access	16 (51.6%)
Transitional piped water access	12 (38.7%)
High piped water access	3 (9.7%)

*IQR* interquartile range

^a^ Average of the daily high temperature for all January days, and separately all July days in the study period (2005–2015) for each province. Days during El Niño events were excluded. The data displayed are the median and IQR of these averaged values for the 194 provinces.

^b^ Refers to the percentage of households in each province for which the primary drinking water source was water piped inside or outside of the home, but within the building area.

^c^ “Low piped water access” provinces were defined as those in which < 60% of households had access to a piped water connection in all study years (2005–2015), or all but 1 year. “High piped water access” provinces were those in which ≥60% of households had access to a piped water connection in all study years, or all but 1 year. “Transitional piped water access” provinces were those that did not fall into either category, i.e., those that transitioned from lower piped water access (< 60% of households with a piped water connection) to higher piped water access (≥60% of households with a piped water connection) between 2005 and 2015.

Long-term diarrhea trends in this dataset have been previously described [8]. Briefly, there were 28.8 annual clinic visits for diarrhea per 100 children < 5 years old in 2005 in Peru, and the rate generally decreased throughout the study period. There was a decline of 3.2% per year in the incidence of childhood clinic visits for diarrhea, controlling for other variables in the model (the weekly mean of daily high temperatures at 1-week, 2-week, and 3-week lags, the rotavirus vaccine era, dry/wet season, moderate/strong El Niño events, and province, Table 2).

**Table 2:** Association between meteorological factors and incidence rate of childhood clinic visits for diarrhea, Peru, 2005–2015

	IRR (95% CI)
Temperature across 3 weeks prior to diarrhea cases^a^	1.038 (1.032, 1.044)
1-week temperature lag^b^	1.014 (1.011, 1.017)
2-week temperature lag^b^	1.016 (1.013, 1.019)
3-week temperature lag^b^	1.008 (1.005, 1.010)
Moderate/strong El Niño period	1.026 (1.009, 1.044)
Dry season	1.014 (1.002, 1.027)
Rotavirus vaccine era (2010–2015)^c^	0.913 (0.886, 0.941)
Year (secular trend)^d^	0.968 (0.961, 0.974)

*IRR* incidence rate ratio, *CI* confidence interval

Multivariable model: IRRs are controlled for other variables in the model/table, and for province. This model also included terms for the interaction between the rotavirus vaccine era and temperature variables; the overall interaction was not significant (*p* = 0.37), thus the temperature estimates stratified by vaccine era are not presented.

^a^ Combined effect of temperature across 3 weeks prior to weekly diarrhea report.

^b^ The 1-week temperature lag is the effect of temperature in the week before the diarrhea cases, the 2-week lag refers to the week before that, etc.

^c^ Compared to the pre-rotavirus vaccine era (2005–2009).

^d^ Continuous term for year.

Nationally, an increase of 1 °C in temperature across the 3 weeks prior to diarrhea cases was associated with a 3.8% higher rate of childhood clinic visits for diarrhea (IRR: 1.038, 95% confidence interval (CI): 1.032–1.044); the individual 1-, 2-, and 3-week temperature lags were each also associated with a higher incidence rate of childhood diarrhea clinic visits (Table 2). Controlling for temperature, there was a significantly higher rate of clinic visits for diarrhea during moderate/strong El Niño events (IRR: 1.026, 95% CI: 1.009–1.044). There was also a modest but significantly higher diarrhea rate in the dry season (IRR: 1.014, 95% CI: 1.002–1.027). In the pre-vaccine era, an increase of 1 °C in temperature across the 3 weeks prior to diarrhea cases was associated with a 3.6% higher childhood diarrhea rate; in the post-vaccine era it was 4.0%. There was not a significant difference in the temperature-diarrhea relationship from the pre- to post-vaccine era (*p* = 0.37).

Generally, coastal provinces had higher levels of access to piped drinking water, with lowest coverage in the Peruvian Amazon provinces (Table 1; Additional File 4). Beginning in 2007, the childhood diarrhea rate was consistently lower in provinces with high piped drinking water access, compared to provinces with low access (Additional File 5). We have previously reported on the association between piped water access and the rate of childhood clinic visits for diarrhea in Peru [8], and here consider access to piped water as a potential modifier of the temperature-diarrhea association.

When analyses were stratified based on access to piped water, the increase in the incidence of childhood clinic visits for childhood diarrhea per 1 °C temperature increase was smaller in the 64 provinces with consistently low access to piped water. In these provinces, an increase of 1 °C in the temperature across the 3 weeks prior to diarrhea cases was associated with a 1.1% higher rate of childhood clinic visits for diarrhea, compared to a 4.3% higher incidence in high piped water access provinces (Table 3). Transitional piped water access provinces displayed similar patterns to high piped water access provinces.

**Table 3:** Associations between meteorological factors and incidence rate of childhood clinic visits for diarrhea, Peru, 2005–2015, stratified by piped water access

	Piped water access^a^		
	Low provinces (*N* = 64) IRR (95% CI)	High provinces (*N* = 56) IRR (95% CI)	Transitional provinces (*N* = 74) IRR (95% CI)
Temperature across 3 weeks prior to diarrhea cases^b^	1.017 (1.007, 1.027)	1.043 (1.034, 1.051)	1.042 (1.033, 1.051)
1-week temperature lag^c^	1.004 (1.000, 1.009)	1.021 (1.016, 1.026)	1.013 (1.008, 1.019)
2-week temperature lag^c^	1.008 (1.003, 1.014)	1.016 (1.011, 1.021)	1.019 (1.014, 1.024)
3-week temperature lag^c^	1.004 (0.999, 1.009)	1.005 (1.000, 1.010)	1.009 (1.004, 1.013)
Moderate/strong El Niño period	1.042 (1.008, 1.077)	1.023 (1.000, 1.046)	1.026 (0.996, 1.057)
Dry season	1.036 (1.014, 1.059)	1.007 (0.989, 1.026)	1.012 (0.992, 1.032)
Rotavirus vaccine era (2010–2015)^d^	0.955 (0.895, 1.019)	0.892 (0.851, 0.934)	0.901 (0.862, 0.942)
Year (secular trend)^e^	0.974 (0.962, 0.985)	0.957 (0.946, 0.968)	0.970 (0.960, 0.981)

*IRR* incidence rate ratio, *CI* confidence interval

Multivariable model: IRRs are controlled for other variables in the model/table, and for province.

^a^ “Low piped water access” provinces were defined as those in which < 60% of households had access to a piped water connection in all study years (2005–2015), or all but 1 year. “High piped water access” provinces were those in which ≥60% of households had access to a piped water connection in all study years, or all but 1 year. “Transitional piped water access” provinces were those that did not fall into either category, i.e., those that transitioned from lower piped water access (< 60% of households with a piped water connection) to higher piped water access (≥60% of households with a piped water connection) between 2005 and 2015.

^b^ Combined effect of temperature across 3 weeks prior to weekly diarrhea report.

^c^ The 1-week temperature lag is the effect of temperature in the week before the diarrhea cases, the 2-week lag refers to the week before that, etc.

^d^ Compared to the pre-rotavirus vaccine era (2005–2009).

^e^ Continuous term for year.

In all piped water access groups, there was a higher incidence of childhood clinic visits for diarrhea during moderate/strong El Niño events, controlling for temperature and other variables in the model. This effect was stronger in provinces with consistently low piped water access. In these provinces, moderate/strong El Niño events were associated with a 4.2% higher childhood diarrhea incidence (IRR: 1.042, 95% CI: 1.008–1.077). In high and transitional piped water access provinces, the association was somewhat lower (2.3 and 2.6%, respectively). There was a higher incidence of childhood clinic visits for diarrhea in the dry season in provinces with low piped water access (IRR: 1.036, 95% CI: 1.014–1.059); there was no significant difference in diarrhea rates between wet and dry seasons in high and transitional piped water access provinces.

In the sensitivity analysis (Additional File 6), the temperature-diarrhea association was somewhat stronger when considering only provinces with higher temperature variability (IRR: 1.045, 95% CI: 1.038–1.052). There was also less of a difference in the temperature-diarrhea association between low piped water access provinces (IRR: 1.034, 95% CI: 1.018–1.050) and high piped water access provinces (IRR: 1.044, 95% CI: 1.034–1.054).

## Discussion

We found a positive association between ambient temperature and childhood clinic visits for diarrhea in Peru (controlling for El Niño events), and also found that El Niño events are associated with more childhood clinic visits for diarrhea even when controlling for temperature. These results are consistent with global research [18], as well as research in Peru [9, 13]. The overall increase in the incidence of childhood clinic visits for diarrhea associated with a 1 °C increase in temperature (3.8%) is in line with a previous global estimate of 7% (95% CI: 3-10%) [18], but is lower than other findings specific to Peru (8–11%) [9, 13]. Other research on the association between temperature and diarrhea in Peru was conducted in Lima in the 1990s, a decade characterized by two El Niño events that were associated with cholera epidemics [9, 13, 15]. The temperature-diarrhea association, and association between El Niño seasons and diarrhea, may be less pronounced in the absence of epidemic cholera, and outside of Lima.

We hypothesized that improved access to piped water might interrupt some of the pathways through which temperature influences diarrhea and dampen the temperature-diarrhea association. Contrary to this hypothesis, the association between temperature and clinic visits for childhood diarrhea was weaker in areas with lower levels of access to piped water, although these differences were not apparent when limiting analyses to provinces with higher temperature variability. A single piped water access category was assigned to each province. Within provinces with higher access to piped drinking water, it is possible that districts with lower piped water access may contribute substantially to the childhood diarrhea burden of that province. On a province level, this could potentially mask any mitigating effects that increased access to piped water may have on the excess childhood diarrhea burden that is associated with higher temperatures. Many of the provinces with low piped water access are geographically large provinces in the Peruvian Amazon region of the country. There may be more exposure misclassification in temperature estimates in these provinces, given their large surface areas. This may bias the association between temperature and clinic visits for child diarrhea toward the null. Also contrary to what we hypothesized, there was no overall difference in the temperature-diarrhea association from the pre- to post-rotavirus vaccine era.

The positive associations between childhood clinic visits for diarrhea and El Niño events, as well as the dry season, were strongest in areas with low piped water access. These areas also tended to have lower access to toilets connected to the sewerage system (Additional File 7). A supplemental analysis showed similar results when instead considering access to sewerage, i.e., the associations between childhood diarrhea and El Niño events and the dry season were also stronger in areas with low sewerage access (Additional File 8). Other research in Peru has found increased risk of diarrhea in children > 5 years old during El Niño in households lacking a sewerage connection, but not in households that do have a sewerage connection [10]. The authors suggest that these children may be more susceptible to the effects of El Niño when they leave their homes to defecate. It is also possible that in the absence of piped water, worse hand hygiene is practiced, leaving children more vulnerable to infections with pathogens circulating during dry and El Niño seasons.

In the Peruvian Amazon region of Peru, El Niño events are associated with lower rainfall and have triggered droughts [39, 40]. Similarly, drought conditions have been associated with some El Niño events in water-scarce southern Peru, at the base of the Andean Mountains [41]. Notably, these are the areas in which many of the provinces with low access to piped water are located (Additional File 4), where diarrhea rates had the strongest association with El Niño events and the dry season. Dry conditions may therefore be a risk factor for childhood diarrhea in Peru, although we did not assess precipitation or drought specifically. Mechanisms through which drought or low rainfall conditions can pose a risk of diarrheal disease have been previously enumerated [19]. In brief, dry conditions may lead to accumulation/increased concentration of fecal pathogens in water and on household surfaces [19, 34]. It is also possible that people travel further distances to obtain drinking water in the dry season, leaving drinking water susceptible to contamination between the source and consumption [19, 42]. The main source of drinking water may also change between seasons, especially if certain communities rely on rainwater during the rainy season and switch to an unimproved source in the dry season. Lack of water may also change handwashing behaviors during the dry season [43]. Compared to research on temperature and heavy rainfall/flooding, there has been less attention on the effects of drought on diarrhea [19], though further consideration of this may be relevant in this setting.

This research was subject to several limitations. We utilized an ecologic study design, which is subject to bias and limits causal inference. Interpretation of analyses that compare provinces with low and high access to piped drinking water are limited by possible spatial confounding, which we did not evaluate. Provinces with low access to piped drinking water tended to be geographically larger, have lower population densities, and located in the Peruvian Amazon region.

We assigned a single weekly temperature value to each province, which involved averaging temperature estimates over both space and time. Some provinces were geographically large, especially in the Peruvian Amazon region. Like provinces, districts tend to be geographically much larger in the Peruvian Amazon region. Notably, many of the provinces that were geographically large had rather homogenous temperature estimates within them, so the choice of averaging the temperature in these provinces may have led to less exposure misclassification than in smaller provinces on the border of the Andean Mountains that have high within-province temperature differences due to differences in elevation (Additional File 9). However, imprecise temperature estimates may have biased our temperature-diarrhea association toward the null.

This analysis did not utilize precipitation data, rather it used more general classifications of the wet and dry season. Wet/dry season was defined based on whether a province was in the coast, mountain, or Peruvian Amazon; however, we used definitions of these three regions that were previously established at the department level, assigning all provinces within each of the 25 departments of Peru to either wet or dry season.

We did not have data on the accessibility of healthcare clinics and how healthcare utilization varied between locations and over time. Areas with lower access to piped drinking water may also have had lower levels of access to clinics and/or unique obstacles in reaching clinics during different seasons. As this analysis focuses on trends in clinic visits for childhood diarrhea, it may not be directly comparable to analyses of the incidence of childhood diarrhea that include cases managed outside of a clinic setting.

## Conclusions

We utilized extensive spatially-detailed weekly data from the country of Peru and found that higher ambient temperatures were associated with significantly higher rates of childhood clinic visits for diarrhea, despite low temperature variability in several provinces. Namely, an increase of 1 °C in the temperature across the three prior weeks was associated with a 3.8% higher rate of childhood clinic visits for diarrhea, with the strongest associations observed at lags of one and 2 weeks, but remaining significant at a three-week lag. The positive associations between temperature and childhood diarrhea rates were, surprisingly, stronger in provinces with higher access to piped water. Moderate/strong El Niño events and the dry season were significantly associated with higher diarrhea rates, controlling for temperature, in provinces with low access to piped water. Unlike earlier research on this topic in Peru, these associations are demonstrated in a decade that did not encompass major cholera epidemics concurrently with El Niño events, and in eras both with and without rotavirus vaccination ongoing. Thus, with data that reflect more recent conditions in Peru (such as ongoing rotavirus vaccination and higher access to piped drinking water), we demonstrate that rising temperatures and intensifying El Niño events pose a risk to child health. Such analyses can be useful in informing vulnerability assessments and mitigation strategies for the effects of climate change on childhood diarrhea.

## Supplementary Information

**Additional File 1.** Comparison of model fit for different temperature variables. Table comparing model fit for temperature variables considered for models.

**Additional File 2.** Weekly mean of daily high temperatures, moderate/strong El Niño periods, and dry seasons, Lima province, Peru, January 2005–December 2015. Time series plot of daily high temperatures for a province in Peru from 2005 to 2015.

**Additional File 3.** Provinces of Peru with high and low annual temperature variability. A map of the provinces of Peru, indicating those with higher and lower maximum temperature variability, as used in a sensitivity analysis.

**Additional File 4.** Piped drinking water access, provinces of Peru, 2005–2015. Map of Peru indicating provinces with varying levels of access to piped water.

**Additional File 5.** Annual rate of clinic visits for childhood diarrhea in Peru, by level of access to piped water, 2005–2015. Figure of the annual rate of clinic visits for childhood diarrhea in Peru, by level of access to piped water, 2005–2015.

**Additional File 6.** Association between meteorological factors and incidence rate of childhood clinic visits for diarrhea, controlling for rotavirus vaccination and secular trend, 61 provinces of Peru with higher temperature variability, by piped water access, 2005–2015. Sensitivity analysis table, provinces with higher temperature variability.

**Additional File 7.** Sewerage access, provinces of Peru, 2005–2015. Map of Peru indicating provinces with varying levels of access to a toilet connected to the sewerage system.

**Additional File 8.** Associations between meteorological factors and childhood diarrhea clinic visit incidence, Peru 2005–2015, stratified by sewerage access. Table of the associations between meteorological factors and childhood diarrhea clinic visit incidence, stratified by sewerage access.

**Additional File 9.** Sample gridded map of the estimated daily high temperature in provinces of Peru. Sample gridded map of the estimated daily high temperature in provinces of Peru, using the PISCOt product.

## Data Availability

Peruvian national census data are available at https://www.inei.gob.pe/estadisticas/censos/. SISFOH data are available at http://iinei.inei.gob.pe/microdatos/ under “Consulta por Encuestas” > “Mapa de Pobreza”. Temperature data (PISCOt product) are publicly available and can be accessed by the IRI/LDEO Climate Data Library at http://iridl.ldeo.columbia.edu/SOURCES/.SENAMHI/.HSR/.PISCO/.Temp/. Oceanic Niño Index data are available at https://psl.noaa.gov/data/climateindices/list/. Ministry of Health data and full study datasets are available from the corresponding author on reasonable request and with permission from the Peruvian Ministry of Health.
