# Supplementary material for: Meteorological factors and childhood diarrhea in Peru, 2005–2015: a time series analysis of historic associations, with implications for climate change
Source: Environ Health. 2021 Feb 26;20:22. doi: 10.1186/s12940-021-00703-4 (PMC7913169; doi:10.1186/s12940-021-00703-4)
Supplement: Supplementary file 1 — Additional File 1. Comparison of model fit for different temperature variables. Table comparing model fit for temperature variables considered for models. [file 12940_2021_703_MOESM1_ESM.docx]

**Additional File 1.** Comparison of model fit for different temperature variables

Three weekly province-level temperature variables were compared for model fit: (1) the weekly maximum of the daily high temperatures, (2) the weekly average of the daily high temperatures, and (3) the weekly average of the daily mean temperatures. We ran three negative binomial generalized estimating equation (GEE) models using each of the three temperature variants above for the week before the diarrhea data were recorded (“1 week lag”), as well as the 2- and 3- week lagged temperatures. Also included in models were: an indicator for the rotavirus vaccine era; an indicator for dry/wet season; an indicator for moderate/strong El Niño events; a continuous variable for the study year; and a variable to control for province.

We found the model with the weekly mean of the daily high temperatures to have the best fit, based on lowest QIC value. In this model, the association between temperature and diarrhea was highest for the 2-week lagged value, but was of similar magnitude for the 1-week lag. After a 2-week lag, the association began to drop off, but was still significant at a 3-week lag. Further analyses considered only the weekly mean of daily high temperatures.

| **Description** | **QIC** |
| --- | --- |
| Weekly average of daily mean temperatures | -55,233,362.89 |
| Weekly average of daily high temperatures | -55,262,848.92 |
| Weekly maximum of daily high temperatures | -55,245,730.09 |
