# Supplementary material for: Meteorological factors and childhood diarrhea in Peru, 2005–2015: a time series analysis of historic associations, with implications for climate change
Source: Environ Health. 2021 Feb 26;20:22. doi: 10.1186/s12940-021-00703-4 (PMC7913169; doi:10.1186/s12940-021-00703-4)

**Additional File 2.** Weekly mean of daily high temperatures, moderate/strong El Niño periods, and dry seasons, Lima province, Peru, January 2005-December 2015


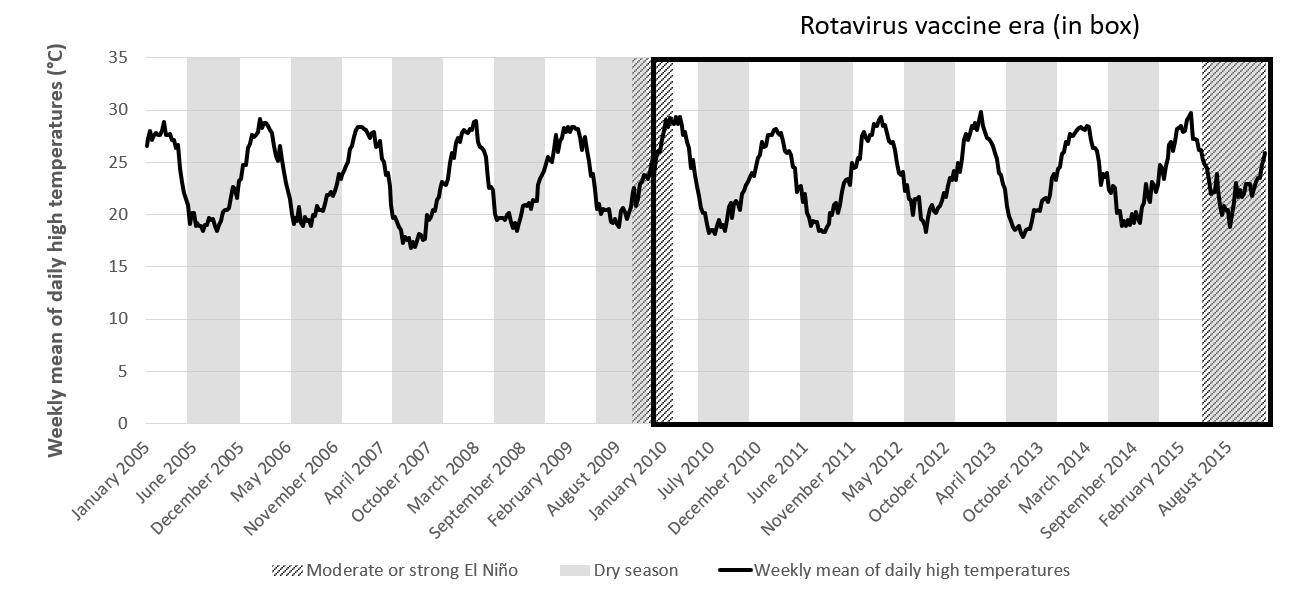

Supplement: Supplementary file 2 — Additional File 2. Weekly mean of daily high temperatures, moderate/strong El Niño periods, and dry seasons, Lima province, Peru, January 2005–December 2015. Time series plot of daily high temperatures for a province in Peru from 2005 to 2015. [file 12940_2021_703_MOESM2_ESM.docx]
