# Supplementary material for: Meteorological factors and childhood diarrhea in Peru, 2005–2015: a time series analysis of historic associations, with implications for climate change
Source: Environ Health. 2021 Feb 26;20:22. doi: 10.1186/s12940-021-00703-4 (PMC7913169; doi:10.1186/s12940-021-00703-4)
Supplement: Supplementary file 3 — Additional File 3. Provinces of Peru with high and low annual temperature variability. A map of the provinces of Peru, indicating those with higher and lower maximum temperature variability, as used in a sensitivity analysis. [file 12940_2021_703_MOESM3_ESM.docx]

**Additional File 3.** Provinces of Peru with high and low annual temperature variability


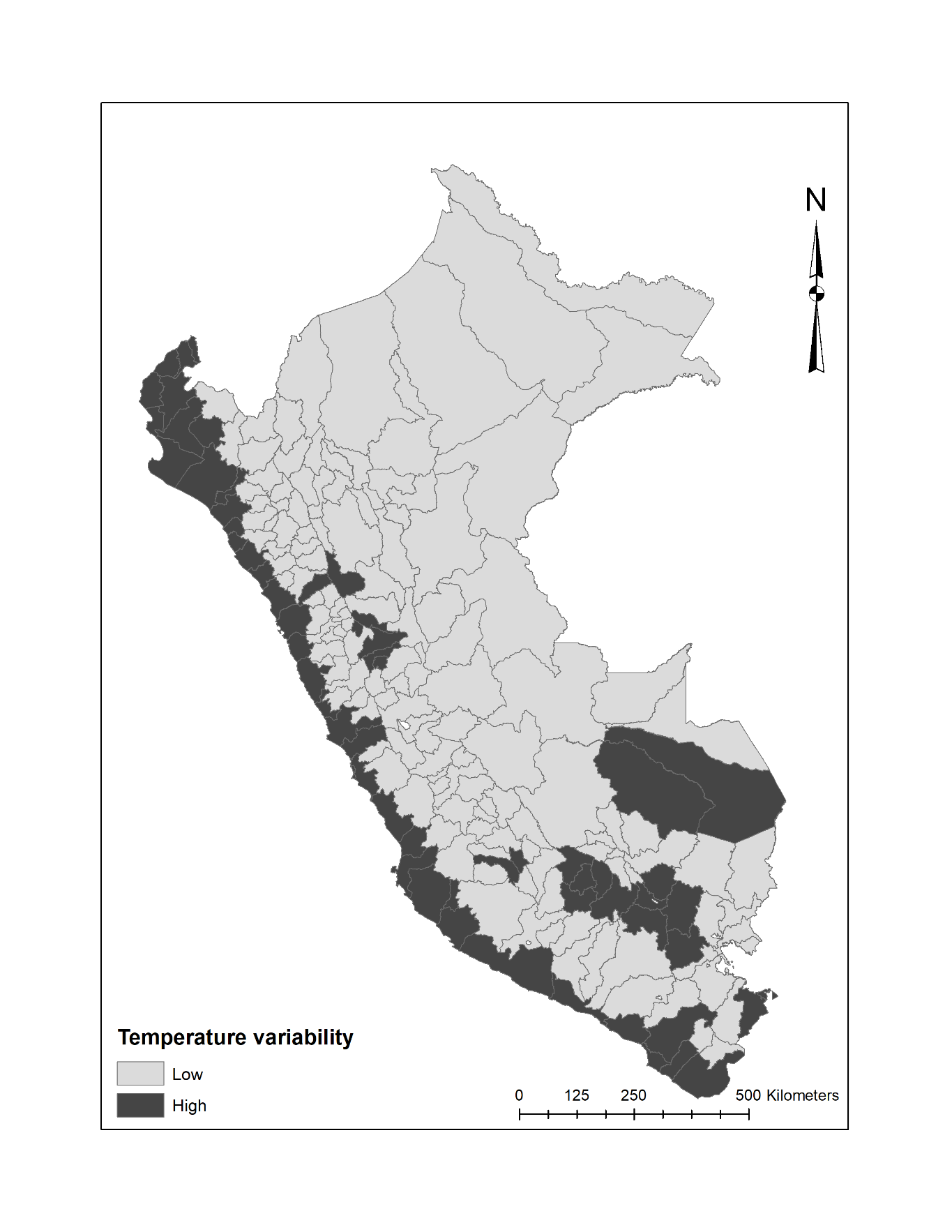


Provinces with high temperature variability are defined as those in which the average daily high temperature in the warmest month of the year was at least 3 °C higher than it was in the coolest month of the year; other provinces are considered to have low variability in maximum temperature.
