# Supplementary material for: Meteorological factors and childhood diarrhea in Peru, 2005–2015: a time series analysis of historic associations, with implications for climate change
Source: Environ Health. 2021 Feb 26;20:22. doi: 10.1186/s12940-021-00703-4 (PMC7913169; doi:10.1186/s12940-021-00703-4)
Supplement: Supplementary file 4 — Additional File 4. Piped drinking water access, provinces of Peru, 2005–2015. Map of Peru indicating provinces with varying levels of access to piped water. [file 12940_2021_703_MOESM4_ESM.docx]

**Additional File 4.** Piped drinking water access, provinces of Peru, 2005-2015


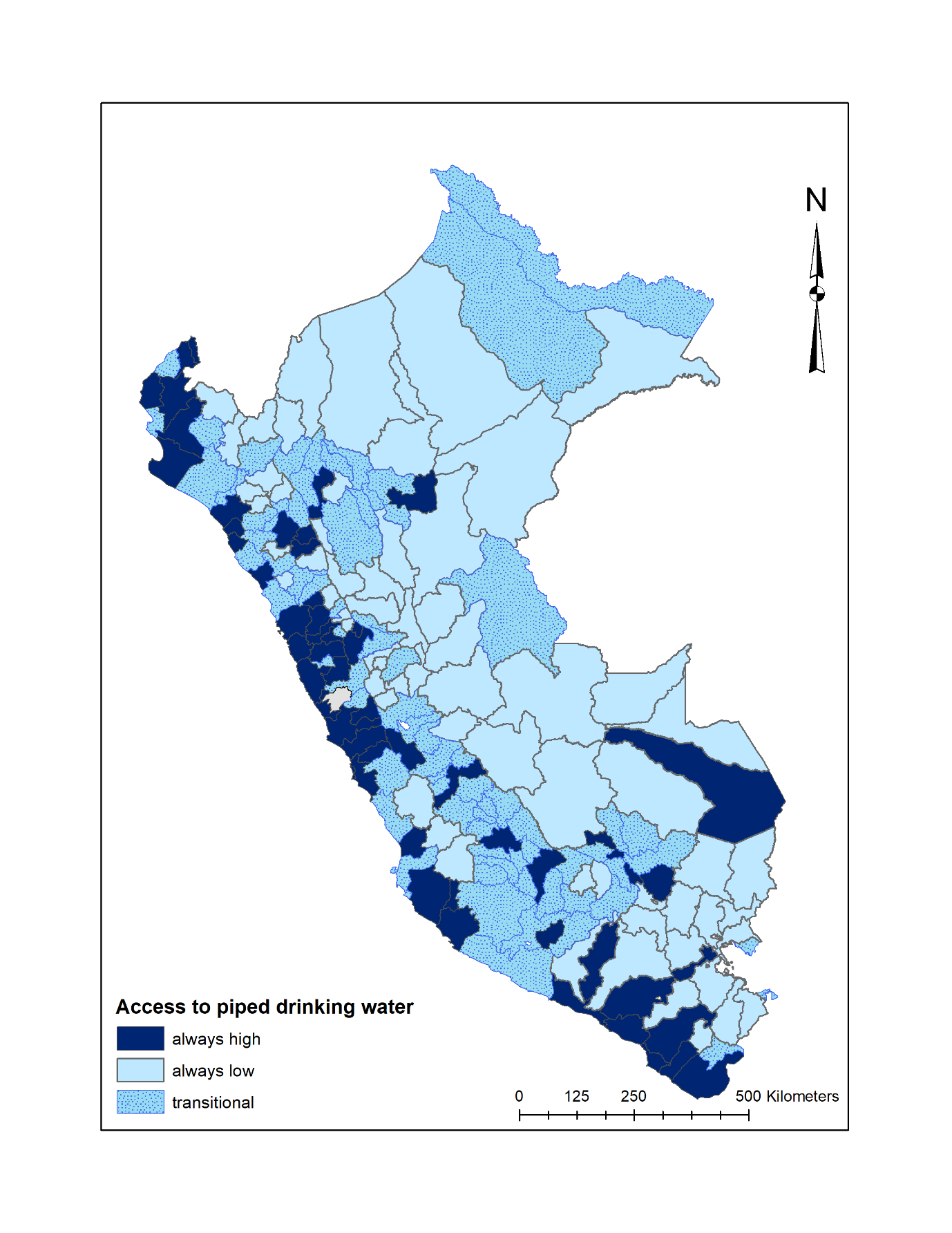


“Always high” water access refers to provinces in which ≥60% of households had access to piped drinking water for every year (or all but one year) from 2005-2015. “Always low” water access refers to provinces in which <60% of households had access to piped drinking water for every year (or all but one year) from 2005-2015. “Transitional” provinces were those that did not fall into either category, *i.e.*, those that transitioned from lower piped water access (<60% of households with a piped connection) to higher water access (≥60% of households with a piped water connection) between 2005 and 2015. Statistics on piped drinking water access from the Peruvian National Institute of Statistics and informatics (Instituto Nacional de Estadística e Informática). Province boundaries obtained from the Permanent Coordinating Committee of the Spatial Data Infrastructure of Peru (Comité Coordinador Permanente de la Infraestructura de Datos Espaciales del Perú).
