# Supplementary material for: Meteorological factors and childhood diarrhea in Peru, 2005–2015: a time series analysis of historic associations, with implications for climate change
Source: Environ Health. 2021 Feb 26;20:22. doi: 10.1186/s12940-021-00703-4 (PMC7913169; doi:10.1186/s12940-021-00703-4)
Supplement: Supplementary file 5 — Additional File 5. Annual rate of clinic visits for childhood diarrhea in Peru, by level of access to piped water, 2005–2015. Figure of the annual rate of clinic visits for childhood diarrhea in Peru, by level of access to piped water, 2005–2015. [file 12940_2021_703_MOESM5_ESM.docx]

**Additional File 5.** Annual rate of clinic visits for childhood diarrhea in Peru, by level of access to piped water, 2005-2015


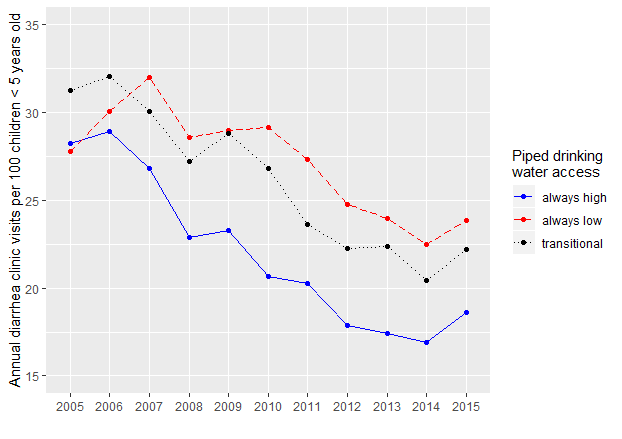


“Always high” water access refers to provinces in which ≥60% of households had access to piped drinking water for every year (or all but one year) from 2005-2015. “Always low” water access refers to provinces in which <60% of households had access to piped drinking water for every year (or all but one year) from 2005-2015. “Transitional” provinces were those that did not fall into either category, *i.e.*, those that transitioned from lower piped water access (<60% of households with a piped connection) to higher water access (≥60% of households with a piped water connection) between 2005 and 2015.
