# Supplementary material for: Meteorological factors and childhood diarrhea in Peru, 2005–2015: a time series analysis of historic associations, with implications for climate change
Source: Environ Health. 2021 Feb 26;20:22. doi: 10.1186/s12940-021-00703-4 (PMC7913169; doi:10.1186/s12940-021-00703-4)
Supplement: Supplementary file 6 — Additional File 6. Association between meteorological factors and incidence rate of childhood clinic visits for diarrhea, controlling for rotavirus vaccination and secular trend, 61 provinces of Peru with higher temperature variability, by piped water access, 2005–2015. Sensitivity analysis table, provinces with higher temperature variability. [file 12940_2021_703_MOESM6_ESM.docx]

**Additional File 6.** Association between meteorological factors and incidence rate of childhood clinic visits for diarrhea, controlling for rotavirus vaccination and secular trend, 61 provinces of Peru with higher temperature variability, by piped water access, 2005-2015

|  | Piped water access^a^ | | |
| --- | --- | --- | --- |
|  | Low provinces (N=11)  IRR (95% CI) | High provinces (N=31)  IRR (95% CI) | Transitional provinces (N=19)  IRR (95% CI) |
| Temperature across three weeks prior to diarrhea cases^b^ | 1.034  (1.018, 1.050) | 1.044  (1.034, 1.054) | 1.047  (1.037, 1.058) |
| 1-week temperature lag^c^ | 1.010  (1.001, 1.019) | 1.025  (1.016, 1.033) | 1.026  (1.019, 1.033) |
| 2-week temperature lag^c^ | 1.015  (1.007, 1.024) | 1.012  (1.006, 1.018) | 1.015  (1.007, 1.024) |
| 3-week temperature lag^c^ | 1.008  (0.998, 1.019) | 1.007  (1.000, 1.014) | 1.006  (0.995, 1.016) |
| Moderate/strong El Niño period | 1.047  (0.985, 1.113) | 0.994  (0.965, 1.024) | 1.005  (0.957, 1.056) |
| Dry season | 1.087  (1.061, 1.113) | 1.017  (0.995, 1.039) | 0.986  (0.944, 1.030) |
| Rotavirus vaccine era (2010-2015)^d^ | 0.948  (0.842, 1.067) | 0.924  (0.871, 0.980) | 0.919  (0.861, 0.981) |
| Year (secular trend)^e^ | 0.951  (0.926, 0.976) | 0.947  (0.932, 0.963) | 0.950  (0.928, 0.974) |

IRR = incidence rate ratio; CI = confidence interval

Multivariable model: IRRs are controlled for other variables in the model/table, and for province.

a.) “Low piped water access” provinces were defined as those in which <60% of households had access to a piped water connection in all study years (2005-2015), or all but one year. “High piped water access” provinces were those in which ≥60% of households had access to a piped water connection in all study years, or all but one year. “Transitional” provinces were those that did not fall into either category, *i.e.*, those that transitioned from lower piped water access (<60% of households with a piped connection) to higher water access (≥60% of households with a piped water connection) between 2005 and 2015.

b.) Combined effect of temperature across three weeks prior to weekly diarrhea report.

c.) The 1-week temperature lag is the effect of temperature in the week before the diarrhea cases, the 2-week lag refers to the week before that, etc.

d.) Compared to the pre-rotavirus vaccine era (2005-2009).

e.) Continuous term for year.
