# Supplementary material for: Meteorological factors and childhood diarrhea in Peru, 2005–2015: a time series analysis of historic associations, with implications for climate change
Source: Environ Health. 2021 Feb 26;20:22. doi: 10.1186/s12940-021-00703-4 (PMC7913169; doi:10.1186/s12940-021-00703-4)
Supplement: Supplementary file 7 — Additional File 7. Sewerage access, provinces of Peru, 2005–2015. Map of Peru indicating provinces with varying levels of access to a toilet connected to the sewerage system. [file 12940_2021_703_MOESM7_ESM.docx]

**Additional File 7.** Sewerage access, provinces of Peru, 2005-2015

**
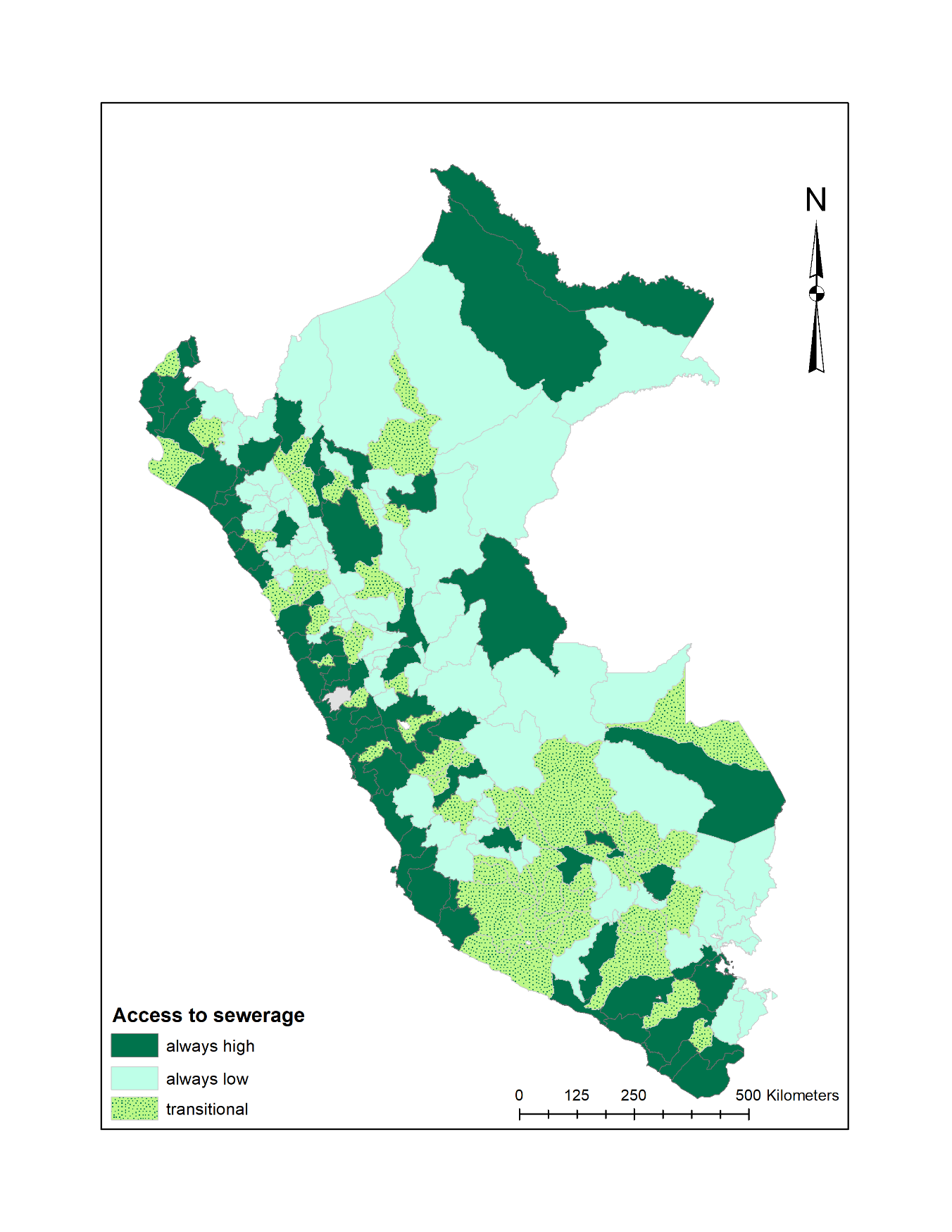
**

“Always high” sewerage access refers to provinces in which ≥30% of households had a toilet connected to the sewerage network for every year (or all but one year) from 2005-2015. “Always low” sewerage access refers to provinces in which <30% of households had a toilet connected to the sewerage network for every year (or all but one year) from 2005-2015. “Transitional” provinces were those that did not fall into either category, *i.e.*, those that transitioned from lower sewerage access (<30% of households with a toilet connected to the sewerage network) to higher sewerage access (≥30% of households with a toilet connected to the sewerage network) between 2005 and 2015. Statistics on sewerage access from the Peruvian National Institute of Statistics and informatics (Instituto Nacional de Estadística e Informática). Province boundaries obtained from the Permanent Coordinating Committee of the Spatial Data Infrastructure of Peru (Comité Coordinador Permanente de la Infraestructura de Datos Espaciales del Perú).
