# Supplementary material for: Meteorological factors and childhood diarrhea in Peru, 2005–2015: a time series analysis of historic associations, with implications for climate change
Source: Environ Health. 2021 Feb 26;20:22. doi: 10.1186/s12940-021-00703-4 (PMC7913169; doi:10.1186/s12940-021-00703-4)
Supplement: Supplementary file 8 — Additional File 8. Associations between meteorological factors and childhood diarrhea clinic visit incidence, Peru 2005–2015, stratified by sewerage access. Table of the associations between meteorological factors and childhood diarrhea clinic visit incidence, stratified by sewerage access. [file 12940_2021_703_MOESM8_ESM.docx]

**Additional File 8.** Associations between meteorological factors and childhood diarrhea clinic visit incidence, Peru 2005-2015, stratified by sewerage access

|  | Sewerage access^a^ | | |
| --- | --- | --- | --- |
|  | Low provinces (N=72)  IRR (95% CI) | High provinces (N=68)  IRR (95% CI) | Transitional provinces (N=54)  IRR (95% CI) |
| Temperature across three weeks prior to diarrhea cases^b^ | 1.022  (1.010, 1.033) | 1.039  (1.032, 1.047) | 1.047  (1.037, 1.058) |
| 1-week temperature lag^c^ | 1.005  (1.000, 1.010) | 1.019  (1.014, 1.024) | 1.015  (1.010, 1.020) |
| 2-week temperature lag^c^ | 1.010  (1.004, 1.015) | 1.015  (1.011, 1.019) | 1.022  (1.016, 1.028) |
| 3-week temperature lag^c^ | 1.007  (1.001, 1.012) | 1.005  (1.001, 1.009) | 1.010  (1.004, 1.015) |
| Moderate/strong El Niño period | 1.049  (1.016, 1.084) | 1.016  (0.992, 1.042) | 1.016  (0.986, 1.046) |
| Dry season | 1.031  (1.011, 1.051) | 1.008  (0.992, 1.025) | 1.014  (0.989, 1.040) |
| Rotavirus vaccine era (2010-2015)^d^ | 0.909  (0.855, 0.967) | 0.918  (0.881, 0.956) | 0.918  (0.869, 0.969) |
| Year (secular trend)^e^ | 0.979  (0.968, 0.991) | 0.957  (0.946, 0.967) | 0.968  (0.958, 0.979) |

IRR = incidence rate ratio; CI = confidence interval

Multivariable model: IRRs are controlled for other variables in the model/table, and for province.

a.) “Low sewerage access” provinces were defined as those in which <30% of households had access to a toilet connected to the sewer system in all study years (2005-2015), or all but one year. “High sewerage access” provinces were those in which ≥30% of households had access to a toilet connected to the sewer system in all study years, or all but one year. “Transitional” provinces were those that did not fall into either category, *i.e.*, those that transitioned from lower sewerage access (<30% of households with access) to higher sewerage access (≥30% of households with a piped water connection) between 2005 and 2015.

b.) Combined effect of temperature across three weeks prior to weekly diarrhea report.

c.) The 1-week temperature lag is the effect of temperature in the week before the diarrhea cases, the 2-week lag refers to the week before that, etc.

d.) Compared to the pre-rotavirus vaccine era (2005-2009).

e.) Continuous term for year.
