# Supplementary material for: Meteorological factors and childhood diarrhea in Peru, 2005–2015: a time series analysis of historic associations, with implications for climate change
Source: Environ Health. 2021 Feb 26;20:22. doi: 10.1186/s12940-021-00703-4 (PMC7913169; doi:10.1186/s12940-021-00703-4)
Supplement: Supplementary file 9 — Additional File 9. Sample gridded map of the estimated daily high temperature in provinces of Peru. Sample gridded map of the estimated daily high temperature in provinces of Peru, using the PISCOt product. [file 12940_2021_703_MOESM9_ESM.docx]

**Additional File 9.** Sample gridded map of the estimated daily high temperature in provinces of Peru


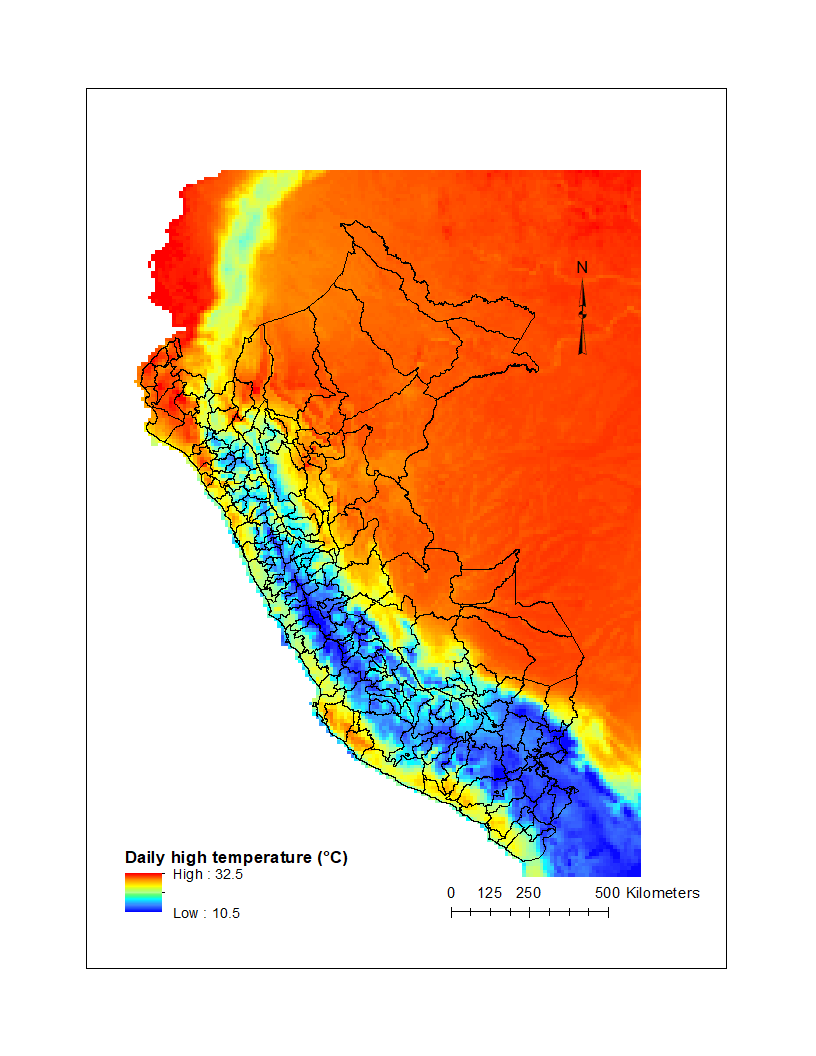


Source: PISCOt product (Peruvian Interpolation data of SENAMHI’s Climatological and hydrological Observations); National Meteorology and Hydrology Service of Peru (SENAMHI) [30]. Sample data for May 15, 2011.
